# NANOTEX BONE Graft along with Fibula Flap in the Reconstruction of Segmental Mandibular Defect: Protocol for Pilot Clinical Trial

**DOI:** 10.29337/ijsp.185

**Published:** 2023-02-10

**Authors:** Anna Serene Babu, R. Rashmi, V. Manju, Pramod Subash, Arjun Krishnadas, Manitha Nair, Subramania Iyer

**Affiliations:** 1 Department of Prosthodontics, Amrita School of Dentistry, Amrita Institute of Medical Science and Research Centre, Kochi, India; 2 School of Nanosciences and Molecular Medicine, Amrita Vishwa Vidyapeetham, Kochi, India; 3 Centre for Cranio-maxillofacial Surgery, Amrita Institute of Medical Science and Research Centre, Kochi, India; 4 Centre for Plastic and Reconstructive Surgery, Amrita Institute of Medical Science and Research Centre, Kochi, India

**Keywords:** Mandibular reconstruction, pilot clinical trial, Nanocomposite fibrous scaffold, Segmental mandibulectomy, Dental implant, ameloblastoma

## Abstract

**Introduction and objectives:**

Mandible reconstruction with vascularized fibula flap is the standard treatment for segmental mandibulectomy in patients with tumor or trauma. But the height of the fibula graft is insufficient for dental implant placement and prosthetic rehabilitation to replace the missing teeth, which in turn will compromise the functional efficiency and aesthetics of the patient. Although the bone height can be augmented through onlay grafting with iliac crest, it is associated with limitations like donor site morbidity and fast resorbability. This suggests the need for a synthetic biomaterial for vertical bone augmentation in implant dentistry.

We have developed a biomimetic, porous, mechanically stable, and biodegradable nanocomposite named “NANOTEX BONE Graft” and its bone regeneration potential was evaluated in pre-clinical animal models. In this clinical trial, the safety as well as the efficacy of NANOTEX to augment new bone over fibula and further its ability to integrate with dental implants will be studied. The study has received the approval of the Ethics Committee of Amrita Institute of Medical Sciences and Central Drugs Standard Control Organization (CDSCO), India.

**Methods:**

We have designed a prospective, single-center, non-randomized pilot clinical study. Patients with benign tumor or trauma indicated for mandibular reconstruction followed by implant rehabilitation will be included in the study. Eligible patients will be enrolled after obtaining informed consent. The study will be initiated and followed up as per defined timelines.

**Highlights:**

- Resection of benign mandibular tumours necessitates surgical removal of jaw bone and adjacent affected areas.
- The segmental mandibulectomy leaves the patient with functional impairments and aesthetic defects which in turn affect the quality of life.
- The standard treatment of reconstruction with vascularized fibula flap has challenge in achieving sufficient vertical bone height for implant placement and prosthetic rehabilitation.
- Alternate surgical techniques cause donor site morbidity and surgical complications.
- There is need for a synthetic biomaterial to be grafted over fibula for vertical bone augmentation.
- NANOTEX BONE Graft, a nanofibrous composite scaffold that mimics native bone, promote cell infiltration, neo-angiogenesis and new bone formation.
- Preclinical studies of NANOTEX in animal models showed bone tissue regeneration, better biodegradation in critical sized defects and efficient integration with dental implants.
- This clinical study propose to evaluate the safety and efficacy of NANOTEX bone graft augmented over fibula in bone regeneration and Titanium dental implant integration.

## 1. Background

### 1.1. Introduction

According to the Union of International Cancer Control Report, the annual incidence of head and neck cancers worldwide is more than 5,50,000, and 90% of this includes the oral cavity [1]. India fact sheet of GLOBOCAN 2020 reports that oral cancers have the second most incidence (10.3%) with 1,35,929 new cases and 8.8% mortality yearly [2]. The International Agency for Cancer Research has predicted that India’s incidence of oral cancer will rise to more than 1.7 million in 2035 [3] due to increased usage of tobacco chewing. Cancer Statistics in India (2018) shows oral cancer as the leading cause of mortality in men, and it is responsible for 25% of cancer-related deaths [4]. World Health Organisation (WHO) report on the global frequency of benign and malignant odontogenic tumors presented that the incidence of tumors affecting the mandible is double that of the maxilla (2.8:1). The benign odontogenic tumors constitute a major percent (95.7%) than the malignant tumors (4.3%), among which ameloblastoma accounts for 30.6% [5]. Treatment of these tumors necessitates surgical removal of the jaw bone, but the resection (either segmental or marginal) leaves the patient with functional impairments like difficulty in mastication, deglutition, and speech along with aesthetic defects like loss of continuity, contour, bone height and width [6].

Vascularised free fibula is the standard autograft used for mandibular reconstruction. Further, Titanium dental implants will be placed to the fibula for dental rehabilitation as it serves as an artificial analog of the missing teeth. This in turn enhances the quality of the patient’s life by improving the masticatory efficiency and aesthetic outcome [7]. Nevertheless, there is a height discrepancy between the native mandible and transplanted fibula, restricting the placement of dental implants [8]. The compromised implant length due to reduced vertical bone height can lead to an improper implant crown root ratio. In addition, an increased prosthetic height to achieve the occlusal plane level will lead to implant overloading and failure of the prosthesis. This necessitates the need for vertical bone augmentation in the fibula reconstructed mandible. Surgical techniques like double-barrelling and vertical distraction osteogenesis can increase bone height, but those techniques are complicated [9]. Onlay grafting using iliac crest (placement of graft on the fibula and its fixation with screws) is another option, but it has drawbacks such as high resorption rate and donor site morbidity [10]. Hence, there is a need for an efficient biomaterial to be placed over fibula, which can promote bone regeneration and increase the bone height. An ideal biomaterial should provide an extracellular matrix microenvironment that mimics the natural bone in regulating cell and tissue behavior. In load-bearing regions like mandible, the strength of the scaffold is also significant in providing signals for bone remodeling. This material also has to accept titanium implant placement. At present, there is no synthetic block graft available for intra-oral bone augmentation purposes to satisfy all these needs.

### 1.2. Rationale

Natural bone is principally a fibrous composite, consisting of inorganic (60–65wt% nanohydroxyapatite (nanoHA) as well as various trace elements like Na, Mg, K, Sr, Zn, Ba, Cu, Fe, and Si) and organic phase (30–35% collagen fibrils). Many synthetic biomaterials like nanoHA, nanoHA composites and Si-HA are widely used in clinics for orthopaedic and dental applications because of their chemical and crystallographic similarity to bone apatite. Still, none of them was found suitable for bone augmentation and Ti dental implantation [11,12,13].

NANOTEX BONE Graft is a fibrous composite that mimics native bone and is made of silica-nanoHA-gelatin with poly L(Lactic acid) (PLLA) fibers [14]. The incorporation of fibers in silica-nanoHA-gelatin matrix can enhance mechanical strength and allow handling during surgical implantation. It is porous (pore size ranges between 50–350 um) and osteoconductive, promoting the infiltration and proliferation of stem cells and osteogenic cells. Besides, it enhances the osteogenic differentiation of mesenchymal stem cells. The material has also been shown to promote the expression of vascular endothelial growth factor, resulting in enhanced endothelial functionality and angiogenesis, a process that is important for new bone formation [15]. Preclinical studies performed in rabbit and pig models proved its ability to promote new bone formation in critical-sized mandible defects through the normal bone healing process. In parallel to bone tissue regeneration, better biodegradation was noted for nanocomposite fibrous scaffold and its biodegradation rate was in par with new tissue formation. More importantly, the newly formed bone could efficiently integrate with Ti dental implants, which was demonstrated in pre-clinical studies [16,17,18].

### 1.3. Study Objectives

The primary objective of this study is to evaluate the safety of the NANOTEX BONE Graft in combination with the fibula flap implanted at segmental mandibulectomy defects. The secondary objective is to evaluate the efficacy of the NANOTEX BONE Graft in bone regeneration when augmented on the fibula and Titanium dental implant integration.

## 2. Methods

### 2.1. Study design

Prospective, single-center, non-randomized pilot clinical study.

### 2.2. Study setting

Amrita Institute of Medical Sciences, Kochi, India. The study treatment duration is expected to be 12 months.

### 2.3. Study intervention

NANOTEX BONE (Nanocomposite Fibrous Scaffold), is a mechanically strong, porous, biodegradable scaffold with bone regeneration properties.

### 2.4. Study participants

Subjects of both genders with 18–65 years of age suitable for mandible reconstruction resulting from tumor resection or trauma.

### 2.5. Eligibility criteria

Inclusion and exclusion criteria are summarized in Table 1.

**Table 1:** Inclusion and exclusion criteria.


INCLUSION CRITERIA	EXCLUSION CRITERIA

Patients must be 18 to 65 years of age while signing the informed consent.	Uncontrolled alcohol, tobacco, or substance abuse within 6 months before implantation

Segmental mandibular defect due to benign tumor resection or trauma	Patients indicated for radiotherapy before and after surgery

A female patient who is neither pregnant nor breastfeeding	Mandibular ramus defects with open wounds

A patient who can report to the study center at defined timelines throughout the study duration	Active uncontrolled infection or malignancy

Patients whose clinical laboratory test results are within the reference range for healthy individuals, or where outside the reference range are judged as not clinically relevant by the Investigator	Systemic disease that would affect the surgical procedure or implant integration including uncontrolled diabetes, osteoporosis, rare bone disorders like osteopetrosis, or any other metabolic bone disease

	Conditions like inherited coagulopathies or bleeding disorders that may affect the implant success or cause post-operative complications

	Increased alkaline phosphatase, increased serum calcium, or Vitamin D deficiency. Oral bisphosphonate or the use of systemic steroids or anabolic agents (e.g., teriparatide) for osteoporosis treatment.

	Patients with inadequate donor sites for lipoaspirate of adipose tissue

	Mixed connective tissue diseases and collagen diseases that can result in poor wound healing after surgery


### 2.6. Sample size

Assuming the level of significance as 5%, the incidence rate of adverse events as 3%, and the margin of error as 10%, the study will be done in 10 subjects to estimate the safety and efficacy with enough precision.

### 2.7. Allocation

Subjects are considered enrolled in the study upon signing the Institute Ethics Committee approved Informed Consent Form (ICF). Informed consent will be obtained before performing any of the study-related procedures or entering any subject data in the Case Report Form. The patient will be given adequate information about the study and sufficient time to comprehend the information in the IC Form before deciding to participate in the study. The study center will be responsible for maintaining subject identification records (Eg. Subject identification log).

### 2.8. Study procedure and data collection

- *NANOTEX BONE graft augmentation on fibula*: A patient who is undergoing segmental mandibulectomy due to benign tumor or trauma will be included in the study after obtaining the consent. Pre-operative planning of the surgery includes virtual surgical planning and model surgery to determine the most ideal position of the fibula and NANOTEX BONE graft. Further, the mandible will be resected followed by free fibula flap reconstruction, which will be stabilized using plates and screws and anastomosed with host vessels as per standard protocol. Further, the height of the fibula will be augmented by placing the scaffold over the fibula and fix with screws and a plate. The size of the block graft will vary depending on the size of the patient defect and fibula. The skin paddle of the fibula flap will be inset to close the mucosal defect over the scaffold and fibula. Post-operative care will be taken as per standard clinical practice for fibula reconstruction.
- *Implant placement and Prosthetic rehabilitation*: The procedure for implant placement involves reconstructed site preparation and implant insertion. The number and size of titanium dental implants will be planned based on bone gain. The loading of the implant (prosthesis placement) will be done if the implant is integrated well with the bone.

### 2.9. Timelines/Participant follow up

Clinical data will be collected at the Screening Visit, Surgery Period (Day 1- Day 10), Follow up Visit (1 month and 3 months), Titanium Implant placement (6 Months), Prosthesis placement (9 months), and End Follow up Visit (12 Months).

### 2.10. Outcome Analysis

#### Primary outcome

*Safety of NANOTEX BONE Graft*: The adverse events will be assessed by 3 parameters: Inflammation, wound dehiscence, and discharging sinus. The assessment will be done at all time points and scores will be given for each category.

#### Secondary outcome

- *Efficacy of NANOTEX BONE graft in supporting Bone Regeneration*: Bone regeneration will be assessed by Cone beam computed tomography (CBCT) assisted by virtual surgical planning and 3D reconstructed stereolithographic models. The radiographic linear measurements will be repeated in the models at each timeline for accuracy and measured in terms of bone height, bone density, bone union, and bone volume during the follow-up scan and its percentage will be measured with the total height.
- *Efficacy in aiding implant prosthetic rehabilitation*: The resonance frequency analysis (RFA) technique will be used to assess Implant Stability Quotient (ISQ) ranging from 1 (lowest stability) to 100 ISQ units (highest stability). The radiological analysis of the bone-implant area will be done and assessed in terms of bone implant contact, peri-implant radiolucency, and crestal bone loss. Soft tissue will be evaluated in terms of soft tissue recession, dehiscence, bleeding on probing and peri-implant exudation.
- *Quality of life of the patient*: The patient’s quality of life will be measured by the Liverpool Oral Rehabilitation Questionnaire-17 (LORQ-17), which assesses issues related to oral function, oro-facial appearance, and social interaction. Oral Health Impact Profile 14 (OHIP 14), which refers to prostheses and patient satisfaction with prostheses will be also rated using a 5-point Likert scale.

### 2.11. Data analysis plan

The sponsor along with Clinical Research Organization (CRO) will conduct the statistical analysis through a separate Statistical Analysis Plan (SAP). In general, continuous data will be presented by descriptive statistics i.e. mean, standard deviation (SD), median, minimum and maximum values. To test the statistical significance between baseline and post findings, paired t-test for normal data or Wilcoxon -Signed rank test for skewed data will be used. Categorical data will be summarized using counts (N: Number of subjects per treatment group, n: number of subjects with non-missing values) and percentages. To test the statistical significance of the changes in the proportion of categorical variables between baseline and post-findings, McNemar’s Chi-Square test will be used.

### 2.12. Quality control

Sponsor/CRO shall ensure proper monitoring of this clinical study through trained clinical research associates or monitors appointed by them. To ensure that the study is conducted following the CIP, the Clinical Trial Agreement, and applicable regulatory and local requirements, the Sponsor/CRO representative or delegates will be allowed access to the subjects’ case histories (clinic and hospital records, and other source data/documentation) upon request. The consent form will be available for monitoring and auditing. The Principal Investigator and site personnel will provide the monitor (s) with complete access to primary source data (e.g., paper and electronic hospital/clinical charts, laboratory records), which support the CRF data and other documents regarding the conduct of the study. Monitoring visits may be scheduled periodically to ensure high degree of data quality.

## Appendix A

**Table:**

DATA CATEGORY	INFORMATION

Trial identification number	CTRI/2022/07/044291

Date of CTRI registration	25/07/2022

Sources of monetary or financial support	Biotechnology Industry Research Assistance Council (BIRAC), Govt. of India

Sponsor	Amrita Vishwa Vidyapeetham, Amrita Institute of Medical Sciences, Kochi, Govt. of India

Contact for public queries	subu.amrita@gmail.com

Contact for scientific queries	manithanair80@gmail.com

Study type	Non-randomized Control Trial

Public title	Safety and efficacy evaluation of NANOTEX BONE graft along with fibula flap in reconstruction of the segmental mandibular defect – A Pilot Clinical Trial

Scientific title	A prospective, single-center, non-randomized pilot clinical study to evaluate the safety and efficacy of biodegradable NANOTEX BONE graft (Nanocomposite Fibrous Scaffold) along with fibula flap in reconstruction of segmental mandibular defect due to tumor resection or trauma

Countries of recruitment	India

Intervention	NANOTEX BONE graft

Date of the first patient enrolled	01/08/2022

Recruitment status	Started recruiting

Key inclusion criteria	Age- 18 to 65 years; Segmental mandibular defect due to benign tumor or trauma resection.

Key exclusion criteria	Patients indicated for radiotherapy before and after surgery; Mandibular ramus defects with open wounds; Active uncontrolled infection or malignancy; systemic diseases affecting surgical outcome

Primary outcome	To evaluate the safety of the NANOTEX BONE Graft in combination with fibula flap for bone augmentation

Secondary outcomes	To evaluate the efficacy of the NANOTEX BONE Graft in combination with fibula flap to regenerate new bone in aiding Titanium dental implant placement and integration

## Trial Registration Number and approval

This trial was approved by the Central Drugs Standard Control Organization (CDSCO) on 17^th^ May 2022 with Permission number: CI/MD/2022/000012The study is registered in the Clinical Trial Registry with the trial number: CTRI/2022/07/044291 (Registered on 25/07/2022) (Protocol Version 1.0 dated 16 August 2021).
